# Synergistic psychosocial pathways linking resilience to self-efficacy in lung cancer following video-assisted thoracic surgery: a cross-sectional study

**DOI:** 10.1016/j.ijnsa.2026.100705

**Published:** 2026-10-11

**Authors:** Xinru Chen, Gaoyang Duan, Jiudi Zhong, Heping Wu, Yang Dong, Dandan Liu

**Affiliations:** a Department of Thoracic Surgery, State Key Laboratory of Oncology in South China, Collaborative Innovation Center for Cancer Medicine, Sun Yat-sen University Cancer Center, Guangzhou, China; b Department of Geriatrics,The Second Affiliated Hospital of South China University, of Technology/ Guangzhou First People's Hospital,Guangzhou, China

**Keywords:** Lung neoplasms, Video-assisted thoracoscopic surgery, Self-efficacy, Resilience, Psychology, Perioperative Nursing

## Abstract

**Background:**

Recovery after video-assisted thoracic surgery (VATS) can involve pain, fatigue, distress, and reduced confidence in rehabilitation activities

**Objectives:**

Guided by conservation of resources theory, this study examined associations between resilience and cancer-related self-care self-efficacy measured during early postoperative recovery, the indirect roles of social support and negative emotion, and whether social support moderated the resilience-self-efficacy association.

**Design:**

A cross-sectional study.

**Settings:**

One tertiary Grade A cancer hospital in South China

**Participants:**

A convenience sample of 225 adults with primary lung cancer who underwent elective VATS was assessed on postoperative days 3 to 5 before discharge.

**Methods:**

Participants completed a sociodemographic/clinical questionnaire and validated Chinese versions of the social support, cancer self-care self-efficacy, resilience, and negative emotion scales. Observed-variable serial mediation paths were estimated with 5000 bootstrap resamples; moderation was examined with a resilience by social support interaction

**Results:**

Resilience was positively associated with self-efficacy (B = 2.080, 95% CI 1.826 to 2.334). The indirect associations through social support (B = 0.118, 95% CI 0.024 to 0.212), negative emotion (B = 0.084, 95% CI 0.004 to 0.164), and both variables sequentially (B = 0.018, 95% CI 0.004 to 0.032) were statistically significant. The resilience by social support interaction was also significant (beta = 0.083, p = 0.010).

**Conclusions:**

In this early postoperative sample, resilience, social support, negative emotion, and self-efficacy showed patterns consistent with the proposed resource-based model. Nursing strategies that assess and support these resources should be tested in longitudinal and interventional studies.

**Contribution of the Paper**

What is already known about the topic

• Lung cancer remains a leading cause of global cancer mortality, even with advancements in minimally invasive thoracoscopic procedures.
• Surgical stress and physical changes after lung resection frequently trigger emotional distress, depression, and anxiety in recovering patients.
• Psychological resilience is an internal resource associated with lower emotional distress and better adjustment in cancer survivors.

What this paper adds

• This study evaluates associations through which social support and negative emotion may link resilience with postoperative self-efficacy.
• The findings identify social support and negative emotion as statistically significant indirect pathways in the observed association between resilience and self-efficacy.
• The results identify candidate targets for perioperative nursing interventions and for prospective intervention research.

## 1 Introduction

Lung cancer remains a leading cause of cancer-related mortality worldwide (Bray et al., 2024; Zhou et al., 2024). Video-assisted thoracoscopic surgery (VATS) is an established minimally invasive approach to lung resection, but recovery still requires physical and psychological adjustment (Pan et al., 2024; Laven et al., 2024). Conservation of resources (COR) theory provides the organising framework for examining this adjustment. The theory proposes that individuals seek to retain, protect, and acquire valued resources, and that threatened or actual resource loss contributes to stress (Hobfoll, 1989). Applied to recovery after VATS, postoperative demands may place pressure on patients' personal and social resources. COR theory also recognises that resources can occur together and that access to external resources may help individuals maintain and use their personal resources (Hobfoll et al., 2018).

Within enhanced recovery after surgery (ERAS) pathways, patients undergoing VATS are encouraged to mobilise early and participate in recovery activities (Ding et al., 2024). Patients therefore need to manage postoperative symptoms while engaging in these activities. Self-efficacy, defined as confidence in one's ability to perform the actions required to manage particular demands, is relevant to engagement in self-care (Bandura, 1977; Bandura, 1998). Research on digital pulmonary rehabilitation has evaluated approaches to supporting physical recovery and psychosocial outcomes after lung cancer surgery (Lu et al., 2024). A latent profile analysis identified distinct postoperative rehabilitation self-efficacy profiles and found associations with resilience, social support, and symptom burden (Chen et al., 2024). These findings support examining confidence in self-care during early recovery after VATS. In the present study, the outcome is cancer-related self-care self-efficacy assessed during this early postoperative period.

Within the COR framework, resilience is conceptualised as a personal psychological resource reflecting the capacity to adapt to adversity (Richardson, 2002). Psychosocial resources related to resilience have been associated with lower psychological distress among cancer survivors (Harms et al., 2019), and a positive association between resilience and self-efficacy has been reported in postoperative patients with non-small cell lung cancer (Chen et al., 2020). Social support is an external resource that can provide emotional reassurance, practical assistance, and information during stressful health experiences (Feeney and Collins, 2015). A resource-based interpretation suggests that patients with greater adaptive capacity may be better able to access and use available support, while supportive relationships may help them maintain confidence in self-care. This provides a rationale for examining the resilience–self-efficacy association and a proposed indirect association through social support.

Negative emotion captures affective experiences that may accompany the demands of cancer and surgery; it is not a direct measure of resource loss. Higher resilience has been associated with lower emotional distress in patients with cancer (Min et al., 2013). Research among women with breast cancer has linked social support to lower anxiety and depression (Zamanian et al., 2021), while research among patients with prostate cancer has reported positive associations between social support and self-efficacy and negative associations between self-efficacy and emotional distress (Wang et al., 2022). These findings support considering emotional experiences alongside personal and external resources. The proposed model therefore examines an indirect association through negative emotion and a sequential indirect association through social support followed by negative emotion. This ordering is a theoretical specification, rather than a temporal sequence established by the available cross-sectional evidence.

COR theory also provides a basis for considering whether personal resources and the surrounding resource environment complement one another (Hobfoll et al., 2018). We therefore hypothesised that the positive resilience–self-efficacy association would be stronger at higher levels of social support. This moderation hypothesis addresses a different question from the indirect-association model: whether the strength of the association varies according to the support environment. The postoperative profile-based and correlational studies described above identify relevant associations (Chen et al., 2024; Chen et al., 2020), but do not resolve the proposed separate and sequential indirect pathways or the moderation question during early recovery after VATS. Addressing these questions could help perioperative nurses identify psychosocial factors to consider together when assessing patients' confidence in self-care.

Guided by this framework, the study aimed to examine the association between resilience and self-efficacy to assess the relevance of a personal psychological resource to confidence in self-care. It also aimed to assess separate and sequential indirect associations through social support and negative emotion, reflecting the proposed connections between resources and emotional experiences. The moderation analysis aimed to evaluate whether the resilience–self-efficacy association varied across levels of social support, addressing the proposed role of the surrounding resource environment. Given the cross-sectional design, these analyses concern statistical associations rather than established temporal or causal processes.

## 2 Methods

### 2.1 Participants and sampling

A cross-sectional study design was employed. Using convenience sampling, we recruited lung cancer inpatients who underwent video-assisted Thoracic Surgery (VATS) at the Department of Thoracic Surgery in a tertiary Grade A cancer hospital in South China between February and August 2025.

The inclusion criteria were as follows: (1) diagnosis of primary lung cancer by pathology or cytology; (2) underwent elective VATS; (3) aged ≥ 18 years; (4) assessed during the acute postoperative recovery phase, specifically postoperative days 3 to 5 before discharge; (5) clear consciousness with the ability to understand and complete the questionnaire; and (6) voluntarily signed the written informed consent form.

The exclusion criteria were as follows: (1) had a history of severe mental illness or cognitive impairment; (2) were transferred to the intensive care unit (ICU) postoperatively or experienced severe postoperative complications, including respiratory failure and severe hemorrhage; (3) had other severe, uncontrolled somatic diseases, including advanced heart failure, that significantly affect quality of life; and (4) had a diagnosis of secondary metastatic lung.

Sample-size planning was guided by the requirements of multivariable regression and bootstrap mediation analysis. Using Green’s rule of thumb for multiple regression (*N* ≥ 50 + *8* *m*, where *m* denotes the number of independent variables), a model including approximately 15 demographic, clinical, and psychological predictors required a minimum sample of 170 participants. To allow for potentially incomplete or invalid questionnaires, the target sample size was set at approximately 215–220 participants.

### 2.2 Data collection and instruments

Data were collected from February through August 2025 with a digital survey platform. Trained members of the research team approached eligible inpatients individually in the thoracic surgery ward on postoperative days 3 to 5 before discharge. The Chinese questionnaire is provided in Supplementary File 1.

**Sociodemographic and Clinical Questionnaire**: A researcher-developed questionnaire was used to collect sociodemographic information, including gender, age, religious belief, residence, marital status, education level, personality type, occupation, medical payment method, and exercise frequency. Clinical information included disease stage, recurrence status, and comorbid chronic disease

**Self-Efficacy**: We used the Chinese version of the Strategies Used by People to Promote Health (SUPPH) scale (Yuan et al., 2015). The 28 items cover positive attitude, stress reduction, and decision-making. Each item is rated from 1 (very little confidence) to 5 (very confident), yielding a total score ranging from 28 to 140. The Chinese version has undergone translation and cultural adaptation and has demonstrated an acceptable dimensional structure and construct validity among patients with cancer (Zhuang et al., 2024; Hu et al., 2023). The SUPPH is a cancer self-management measure rather than a procedure-specific postoperative rehabilitation scale. As it was administered on postoperative days 3–5, scores were interpreted as cancer-related self-care self-efficacy during early recovery. Internal consistency for the total score was high in the present sample (Cronbach’s α = 0.93).

**Resilience:** Resilience was assessed using the Chinese 10-item Connor–Davidson Resilience Scale (CD-RISC-10) (Wang et al., 2010). IItems are rated from 0 to 4, with higher total scores indicating greater resilience. Previous studies have supported the scale’s unidimensional structure and construct validity in Chinese populations (Cheng et al., 2020; Cao et al., 2021). Internal consistency was acceptable in the present sample (Cronbach’s α = 0.786).

**Social Support**: Social support was assessed using the Social Support Rating Scale (SSRS), developed by Xiao. The 10 items assess objective support, subjective support, and support utilization, with higher scores indicating greater available and perceived social support. The total score was calculated according to the original item-specific scoring procedure: Items 1–4 and 8–10 are scored from 1 to 4; Item 5 assesses support from family members; and Items 6–7 assess the number of available support sources. Previous studies have supported the scale’s content and construct validity in Chinese populations (Li et al., 2021; Yu et al., 2015). Internal consistency was high in the present sample (Cronbach’s α = 0.90).

**Negative Emotion**: Negative emotion was assessed using the Negative Affect subscale of the Positive and Negative Affect Schedule (PANAS) (Watson et al., 1988). The subscale contains 10 items rated from 1 to 5, with higher total scores indicating greater negative affect. Participants rated their experiences during the preceding 2 weeks; thus, the score reflects affect across the preoperative and perioperative period rather than affect exclusively after surgery. PANAS has demonstrated acceptable internal consistency and construct validity in Chinese populations (Zhao et al., 2024; Zheng et al., 2016). Internal consistency for the negative-affect subscale was good in the present sample (Cronbach’s α = 0.83).

Following individual invitations, trained research staff provided participants with a QR code and were available to clarify procedural questions without guiding or influencing participant responses. Electronic informed consent was presented on the opening page of the survey; participants were required to explicitly select “Agree” to proceed to the questionnaire. Patients completed the survey electronically on‑ward. Survey administration took 10–15 minutes. All survey data were collected anonymously.

### 2.3 Statistical analysis

Statistical analyses were performed using R software (version 4.3.2). Continuous variables are summarized as means, standard deviations (SDs), and medians, whereas categorical variables are presented as frequencies and percentages. Normality was assessed using skewness and kurtosis, with values between −2 and +2 considered acceptable. Given the sample size, parametric analyses were considered sufficiently robust. Missing data were handled using complete-case analysis. Questionnaires with incomplete data on study variables were excluded before analysis, and no imputation was performed.

Univariate analyses, including independent samples t tests, one-way analysis of variance (ANOVA), and Pearson correlation coefficients, were initially conducted to identify demographic and clinical covariates significantly associated with self-efficacy.

To evaluate the predictive ability of the psychosocial constructs, a two-step hierarchical linear regression was performed using base R functions. To rigorously isolate the incremental variance (ΔR^2^) contributed by the core variables, demographic and clinical covariates identified as significant in univariate analyses were entered into Model 1 to control for baseline confounding. The core psychosocial variables (resilience, social support, and negative emotion) were subsequently entered into Model 2. Multicollinearity among predictors was assessed using the variance inflation factor (VIF) via the *car* package, with values < 5 indicating the absence of problematic collinearity.

Serial multiple mediation was used to examine the prespecified observed-variable path model: resilience → social support → negative emotion → self-efficacy, with direct paths from resilience to negative emotion and self-efficacy and from social support to self-efficacy. The model was estimated with the *lavaan* package. Because this path specification is just-identified (df = 0), global fit indices are mathematically saturated and cannot be used to judge model adequacy. Indirect associations were evaluated with bias-corrected bootstrapping using 5000 resamples(23); a 95% confidence interval excluding zero was considered statistically significant.

Finally, to robustly explore the boundary conditions of the relationship between resilience and self-efficacy, a formal moderation analysis was conducted to test the interaction effect between resilience and social support. To avoid the statistical power loss associated with the artificial categorization of continuous variables, simple slope analyses were plotted at mathematically defined levels of social support (e.g., mean ± 1 SD) to visualize and interpret the conditional effects across varying support contexts. Statistical significance was set at *p* < 0.05 (two-tailed) for all analyses.

## 3 Results

### 3.1 Participant characteristics and univariate analysis

A total of 253 post-VATS lung cancer patients were assessed for eligibility. As shown in Supplementary Figure S1, 28 patients were excluded because they did not meet the inclusion criteria, declined participation, or were unable to complete the survey. The remaining 225 eligible patients completed the questionnaires and were included in the final analysis.

A total of 225 lung cancer patients who underwent VATS were included in this study. As detailed in Table 1, the sample was fairly balanced in terms of sex (54.22% female) and was predominantly older, with the majority being aged 60 years or older (52.00%). Most participants resided in urban areas (72.00%), were married (88.00%), and held medical insurance (63.56%). Clinically, most participants reported an early disease course (84.89%) and no prior recurrence (96.89%), although approximately one-third (33.78%) reported comorbid chronic diseases.

**Table 1:** Demographic characteristics of the participants (N = 225).

Characteristics	Category	Number (n)	Proportion (%)
Gender	Male	103	45.78
	Female	122	54.22
Age (years)	18–35	4	1.78
	35–59	104	46.22
	≥60	117	52.00
Religious Belief	No	202	89.78
	Yes	23	10.22
Residence	Rural	63	28.00
	Urban	162	72.00
Marital Status	Married	198	88.00
	Single	5	2.22
	Divorced	4	1.78
	Widowed	18	8.00
Education Level	Primary school or below	39	17.33
	Junior high school	58	25.78
	High school/Technical school	58	25.78
	College or above	70	31.11
Personality Type	Introverted	28	12.44
	Intermediate	121	53.78
	Extroverted	76	33.78
Occupation	Freelance	54	24.00
	Retired	108	48.00
	Employed	50	22.22
	Other	13	5.78
Medical Payment Method	Full public expense	8	3.56
	Medical insurance	143	63.56
	New rural cooperative	50	22.22
	Commercial insurance	19	8.44
	Self-pay	5	2.22
Exercise Frequency	Almost never	60	26.67
	<1 time/week	32	14.22
	1–3 times/week	48	21.33
	>3 times/week	85	37.78
Disease Stage	Early stage	191	84.89
	Middle stage	31	13.78
	Late stage	3	1.33
Recurrence	No	218	96.89
	Yes	7	3.11
Comorbid Chronic Disease	No	149	66.22
	Yes	76	33.78

### 3.2 Descriptive statistics of the main variables

Descriptive statistics for the primary study variables are presented in Table 2. The mean self-efficacy score was 103.21 (SD = 19.68; range: 45–140; median: 103), indicating a moderate-to-high level among the participants.

**Table 2:** Descriptive statistics of the main variables (N = 225).

Variable	Mean	SD	Min	Max	Median	Skewness	Kurtosis
Self-Efficacy	103.21	19.68	45	140	103	−0.19	−0.50
Resilience	28.23	6.65	8	40	29	−0.52	0.00
Social Support	43.71	7.00	20	61	44	−0.32	−0.07
Negative Emotion	20.84	6.80	10	45	20	0.62	0.16

The mean resilience score was 28.23 (SD = 6.65; observed range, 8–40). The mean social support score was 43.71 (SD = 7.00; observed range, 20–61), and the mean negative-affect score was 20.84 (SD = 6.80; observed range, 10–45). Skewness and kurtosis values for all continuous variables were between −2 and +2; for example, self-efficacy had a skewness of −0.19 and kurtosis of −0.50. These results supported the use of parametric analyses.

### 3.3 Univariate analysis

Univariate analyses identified baseline characteristics associated with postoperative self-efficacy. Patients who were younger (*F* = 5.506, *p* = 0.005), lived in urban areas (*t* = −2.628, *p* = 0.010), had higher educational attainment (*F* = 6.062, *p* < 0.001), or reported an extroverted personality (*F* = 6.760, *p* = 0.001) had higher self-efficacy scores. Employment (*F* = 4.511, *p* = 0.004), more frequent exercise (*F* = 2.674, *p* = 0.048), and absence of comorbid chronic disease (*t* = 2.103, *p* = 0.037) were also associated with higher self-efficacy. These variables were entered as baseline covariates in the hierarchical regression models. Detailed comparisons are provided in Supplementary Table S1.

### 3.4 Hierarchical regression analysis

A two-step hierarchical linear regression analysis was performed to determine the independent predictive value of psychosocial variables on self-efficacy, controlling for significant sociodemographic and clinical characteristics (Table 3). Collinearity diagnostics indicated no severe multicollinearity among the predictors (all the VIFs were < 3.0).

**Table 3:** Hierarchical linear regression analysis for predicting self-efficacy.

Variable	Model 1	Model 2			VIF		
	***B* (95% CI)**	***β***	***p***	***B* (95% CI)**	***β***	***p***	
**Constant**	87.04 (68.58, 105.51)	-	<0.001	41.68 (22.69, 60.66)	-	<0.001	-
**Age**	−2.53 (−8.38, 3.32)	−0.069	0.398	−2.05 (−5.95, 1.86)	−0.056	0.305	1.76
**Residence (Urban)**	5.08 (−1.10, 11.25)	0.258	0.109	3.68 (−0.42, 7.77)	0.187	0.080	1.36
**Education level (ref: Primary school or below)**							
Junior high school	3.37 (−4.11, 10.85)	0.171	0.378	0.50 (−4.47, 5.47)	0.025	0.845	1.66
High school/Technical	5.30 (−2.60, 13.19)	0.269	0.190	1.44 (−3.82, 6.69)	0.073	0.593	1.66
College or above	7.75 (−0.52, 16.02)	0.394	0.068	1.36 (−4.20, 6.92)	0.069	0.632	1.66
**Personality type (ref: Introverted)**							
Intermediate	10.55 (2.84, 18.27)	0.536	0.008	1.23 (−4.28, 6.75)	0.063	0.662	1.31
Extroverted	15.27 (7.16, 23.38)	0.776	<0.001	2.17 (−3.68, 8.02)	0.110	0.469	1.31
**Occupation (ref: Freelance)**							
Retired	−2.00 (−8.88, 4.88)	−0.102	0.569	−0.90 (−5.46, 3.67)	−0.046	0.701	2.33
Employed	3.14 (−4.82, 11.10)	0.160	0.440	2.20 (−3.12, 7.53)	0.112	0.418	2.33
Other	−4.59 (−15.84, 6.66)	−0.233	0.424	−0.44 (−7.93, 7.06)	−0.022	0.910	2.33
**Exercise frequency (ref: Hardly ever)**							
< 1 time/week	3.40 (−4.60, 11.39)	0.173	0.406	2.81 (−2.56, 8.17)	0.143	0.306	1.25
1–3 times/week	6.31 (−0.80, 13.42)	0.320	0.084	3.72 (−1.04, 8.48)	0.189	0.127	1.25
> 3 times/week	7.55 (1.38, 13.72)	0.384	0.017	3.45 (−0.70, 7.61)	0.175	0.105	1.25
**Comorbid disease (Yes)**	−2.94 (−8.32, 2.44)	−0.149	0.285	0.76 (−2.83, 4.36)	0.039	0.677	1.16
**Psychosocial variables (Model 2 only)**							
Resilience	-	-	-	2.05 (1.75, 2.34)	0.691	<0.001	1.51
Social support	-	-	-	0.12 (−0.16, 0.40)	0.042	0.400	1.50
Negative emotion	-	-	-	−0.20 (−0.47, 0.07)	−0.068	0.156	1.30
**Model fit**							
R²	0.198			0.653			
Adjusted R²	0.145			0.625			
F-statistic	3.697			22.835			
ΔR² (vs. Model 1)	-			0.455			

In Model 1, baseline covariates (age, residence, education level, personality type, occupation, exercise frequency, and comorbid chronic disease) accounted for 19.8% of the variance in self-efficacy (R^2^ = 0.198, *F* = 3.697, *p* < 0.001). Adding resilience, social support, and negative emotion in Model 2 increased R^2^ by 0.455 (*p* < 0.001). The full model accounted for 65.3% of the variance (R^2^ = 0.653; adjusted R^2^ = 0.625; *F* = 22.835; *p* < 0.001).

In the full model, resilience had the largest standardized coefficient for self-efficacy (*B* = 2.05, 95% CI [1.75, 2.34], *β* = 0.691, *p* < 0.001).

### 3.5 Serial multiple mediation analysis

The observed-variable serial mediation model was just-identified (χ² = 0.000, df = 0).; hence, the χ²/df ratio could not be computed. The CFI = 1.000, TLI = 1.000, RMSEA = 0.000, and SRMR = 0.000 are typical for a saturated model and do not provide an empirical evaluation of global model fit. Path coefficients and bootstrap confidence intervals are reported in Table 4 and Fig. 1.

**Table 4:** Path coefficients and effect estimates in the serial multiple mediation model.

Path/Effect Description	*B*	SE	*z*	*p*	95% CI
Resilience → Self-Efficacy (*c‘*)	2.080	0.130	16.000	<0.001	1.826, 2.334
Resilience → Social Support (*a₁*)	0.470	0.058	8.103	<0.001	0.357, 0.583
Social Support → Negative Emotion (*a₃*)	−0.190	0.070	−2.714	0.007	−0.327, −0.053
Social Support → Self-Efficacy (*b₁*)	0.250	0.105	2.381	0.017	0.045, 0.455
Resilience → Negative Emotion (*a₂*)	−0.400	0.058	−6.897	<0.001	−0.513, −0.287
Negative Emotion → Self-Efficacy (*b₂*)	−0.210	0.100	−2.100	0.036	−0.406, −0.014
Resilience → Social Support → Self-Efficacy (*a₁*× *b₁*)	0.118	0.048	2.458	0.014	0.024, 0.212
Resilience → Negative Emotion → Self-Efficacy (*a₂*× *b₂*)	0.084	0.041	2.049	0.040	0.004, 0.164
Resilience → Social Support → Negative Emotion → Self-Efficacy (*a₁*× *a₃*× *b₂*)	0.018	0.007	2.571	0.010	0.004, 0.032
*Note: The observed-variable path model was just-identified (χ²= 0.000, df = 0). χ²/df is not estimable; CFI = 1.000, TLI = 1.000, RMSEA = 0.000, and SRMR = 0.000 are expected for a saturated model and are not evidence of empirical model fit.*

**Fig. 1:**
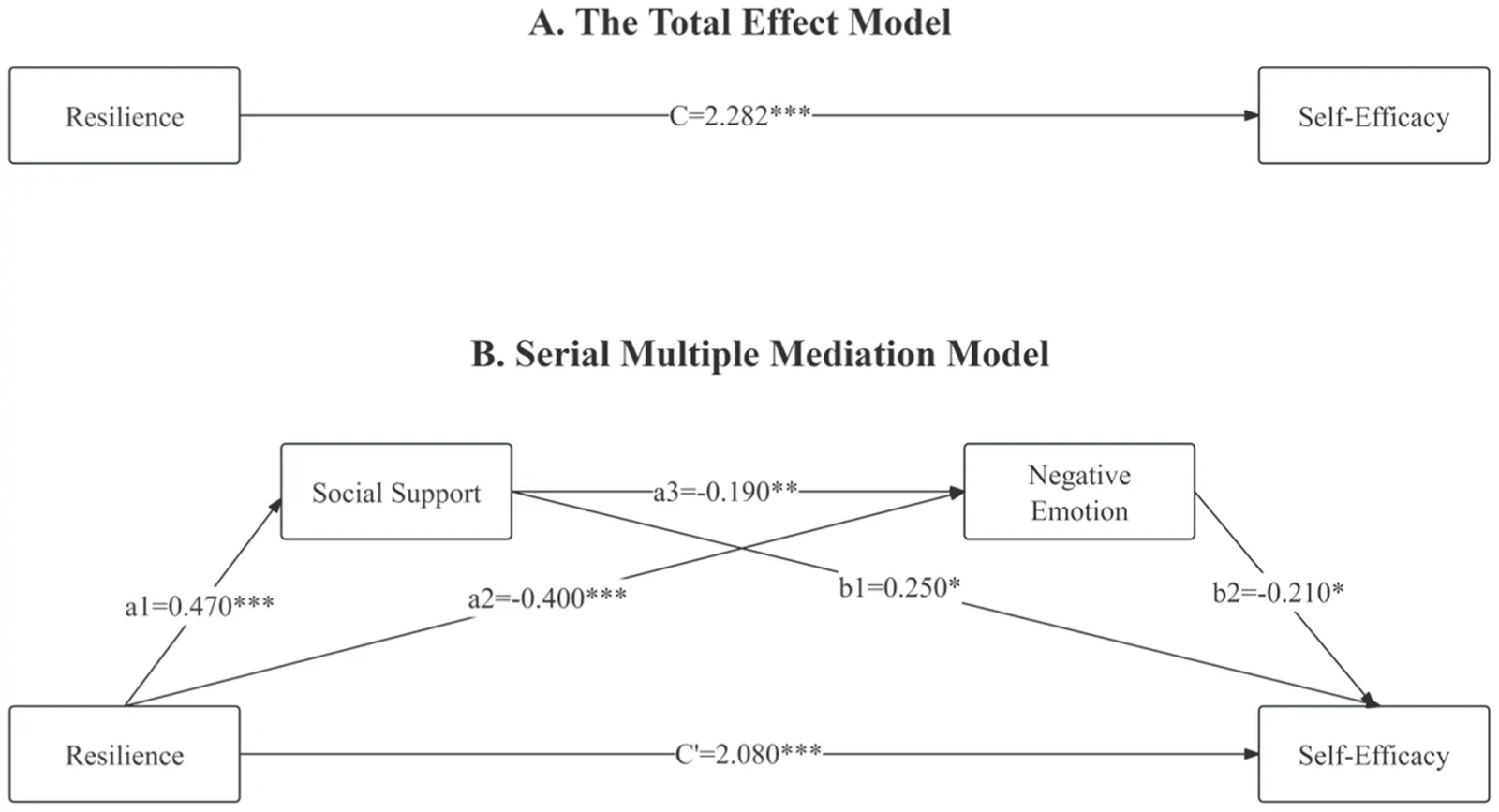
Total effect model (A) and serial multiple mediation model (B) of the effect of resilience on self-efficacy Note: The observed-variable path model was just-identified (χ²= 0.000, df = 0). χ²/df is not estimable; CFI = 1.000, TLI = 1.000, RMSEA = 0.000, and SRMR = 0.000 are expected for a saturated model and are not evidence of empirical model fit. * p *<* 0.05, ** p *<* 0.01, *** p *<* *0.001.*

In the total-effect model, resilience was positively related to self-efficacy (*B* = 2.328, SE = 0.122, *z* = 19.120, *p* < 0.001, 95% CI [2.090, 2.567]). After incorporating social support and negative emotion into the model, the direct effect of resilience on self-efficacy remained statistically significant (*B* = 2.080, SE = 0.130, *z* = 16.000, *p* < 0.001, 95% CI [1.826, 2.334]).

Resilience exerted a positive effect on social support (path *a₁: B* = 0.470, SE = 0.058, *z* = 8.103, *p* < 0.001) and a negative effect on negative emotion (path *a₂: B* = −0.400, SE = 0.058, *z* = −6.897, *p* < 0.001). Social support negatively predicted negative emotion (path *a₃: B* = −0.190, SE = 0.070, *z* = −2.714, *p* = 0.007). Within the mediation model for self-efficacy, social support positively predicted self-efficacy (path *b₁: B* = 0.250, SE = 0.105, *z* = 2.381, *p* = 0.017), whereas negative emotion negatively predicted self-efficacy (path b₂: *B* = −0.210, SE = 0.100, *z* = −2.100, *p* = 0.036).

Bootstrap analyses revealed three statistically significant specific indirect effects linking resilience to self-efficacy. The indirect effect via social support was significant (*a₁* × *b₁*: estimate = 0.118, SE = 0.048, *z* = 2.458, *p* = 0.014, 95% CI [0.024, 0.212]). The indirect effect through negative emotion was also significant (*a₂* × *b₂*: estimate = 0.084, SE = 0.041, *z* = 2.049, *p* = 0.040, 95% CI [0.004, 0.164]). Furthermore, the sequential indirect effect through social support followed by negative emotion reached significance (*a₁* × *a₃* × *b₂*: estimate = 0.018, SE = 0.007, *z* = 2.571, *p* = 0.010, 95% CI [0.004, 0.032]).

Collectively, these results suggest that social support and negative emotion partially mediate the association between resilience and self-efficacy, both independently and in a sequential manner.

### 3.6 Moderating effect of social support

A moderation analysis was performed to test whether social support moderated the relationship between resilience and self‑efficacy. Resilience and social support were mean‑centered prior to constructing the interaction term. The resilience × social support interaction reached statistical significance (*β* = 0.083, SE = 0.032, *t* = 2.594, *p* = 0.010).

Simple‑slope analyses were implemented at low (−1 SD), mean, and high (+1 SD) levels of social support, as illustrated in Fig. 2. Conditional effects of resilience on self‑efficacy were positive across all three levels: *B* = 2.180 (SE = 0.167, *t* = 13.060, *p* < 0.001) for low social support, *B* = 2.221 (SE = 0.136, *t* = 16.320, *p* < 0.001) for mean social support, and *B* = 2.262 (SE = 0.189, *t* = 11.960, *p* < 0.001) for high social support. The positive effect of resilience on self‑efficacy was slightly stronger under higher social support conditions.

**Fig. 2:**
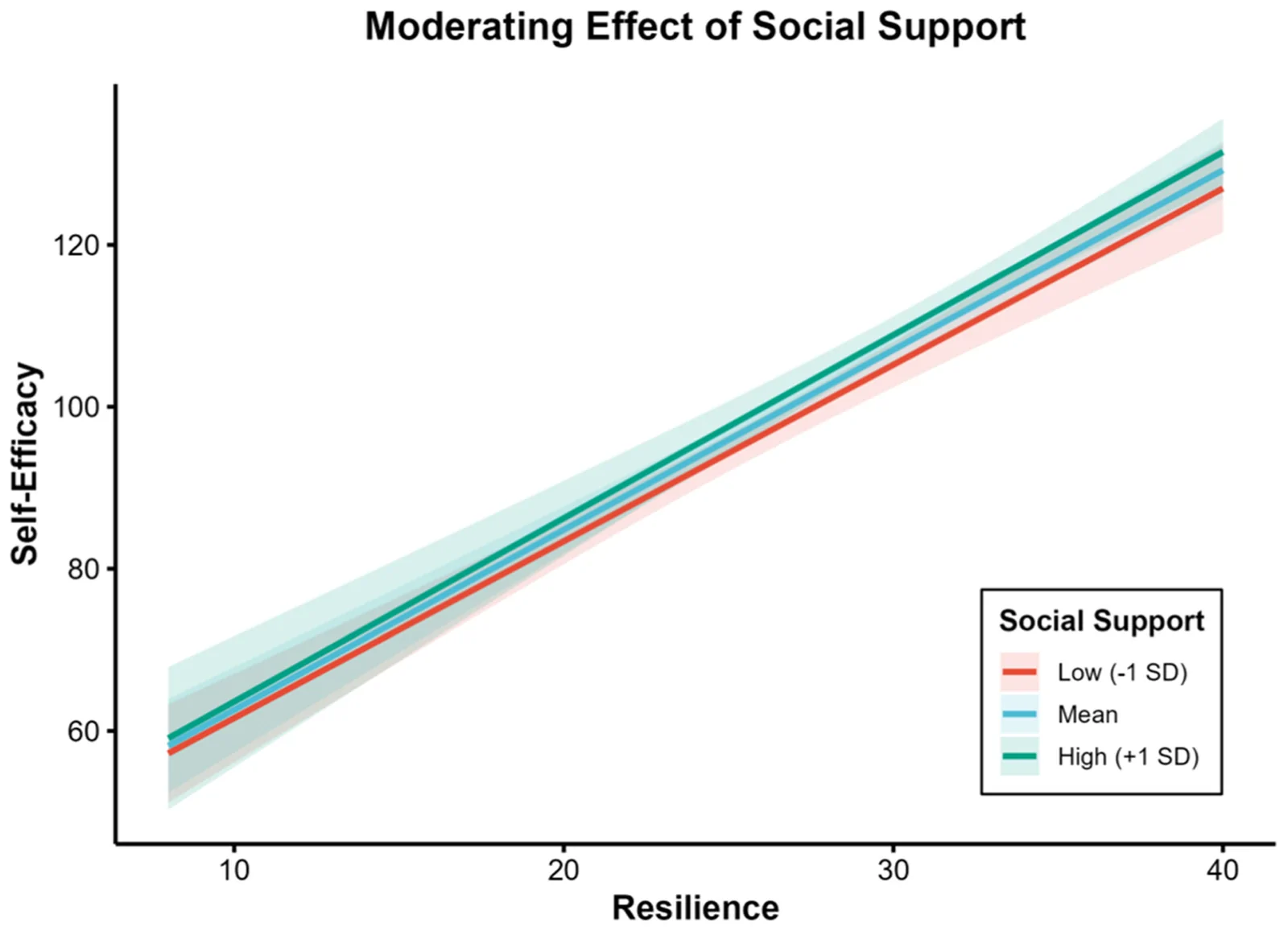
Moderating effect of social support on resilience and self-efficacy Note: Shaded areas represent 95% CIs.

Considering the cross‑sectional nature of the study, this interaction reflects a statistical association rather than causal evidence that elevated social support alters the predictive effect of resilience on self‑efficacy.

## 4 Discussion

This study examined cancer-related self-care self-efficacy during early recovery after VATS. Resilience showed the strongest association with self-efficacy in the adjusted regression model, while the path analyses identified indirect associations involving social support and negative emotion. Social support also moderated the resilience–self-efficacy association. Taken together, these findings place patients’ self-care confidence within a combination of personal adaptive capacity, interpersonal support and emotional experience. COR theory provides a framework for interpreting this pattern because it considers how people retain and draw on valued resources when facing actual or threatened loss (Hobfoll, 1989; Hobfoll et al., 2016). In the present study, this perspective connects resilience as a personal resource with social support as an interpersonal resource, rather than treating either as an isolated explanation for self-efficacy.

The prominent association between resilience and self-efficacy suggests that patients’ capacity to adapt to illness-related demands is relevant to their confidence in managing cancer-related self-care. The additional variance explained after introducing the psychosocial variables (ΔR² = 0.455) represents their collective contribution, not the contribution of resilience alone. Chen et al. similarly reported a positive association between self-efficacy and resilience among postoperative NSCLC patients, although their analysis treated resilience as the outcome (Chen et al., 2020). Chen et al. found that greater resilience and social support were associated with membership in higher postoperative rehabilitation self-efficacy profiles (Chen et al., 2024). These studies converge on the relevance of adaptive resources to confidence, despite differences in analytical direction and outcome definition. Our outcome was cancer-related self-care self-efficacy measured using SUPPH, rather than a procedure-specific measure of rehabilitation confidence. Masten et al.’s multisystem account of resilience also provides a rationale for examining personal adaptive capacity together with the relational resources surrounding patients (Masten et al., 2021).

The indirect associations through social support and negative emotion extend this interpretation beyond personal adaptive capacity. In the specified path model, greater social support and lower negative emotion were each associated with higher self-efficacy. COR theory’s resource-caravan perspective suggests that personal and interpersonal resources tend to occur together (Hobfoll et al., 2018). Supportive relationships may offer practical assistance and reassurance that help patients perceive self-care demands as manageable, an interpretation consistent with Feeney and Collins’ account of support during adversity (Feeney and Collins, 2015). The emotional association has a complementary basis: Alarcón et al. found that resilience was negatively correlated with negative affect after breast cancer surgery (Alarcón et al., 2020), while Min et al. reported lower emotional distress among patients with greater resilience (Min et al., 2013). These comparisons support the relevance of emotional experience, but PANAS negative affect should not be equated with clinical anxiety or depression. Its two-week recall period also spans preoperative and perioperative experiences. The indirect findings should be considered alongside the fully adjusted regression, in which neither social support nor negative emotion retained a statistically significant independent association with self-efficacy.

The serial indirect association brings these relational and emotional findings into a single configuration: greater resilience was associated with greater social support, greater support with lower negative emotion, and lower negative emotion with higher self-efficacy. This configuration is compatible with the COR view that adaptation involves connected resources rather than separate protective attributes. Zamanian et al. found that functional coping partly mediated associations between social support and anxiety or depression in women with breast cancer, supporting the relevance of support to emotional experience (Zamanian et al., 2021). However, the ordering of these relationships differs across studies. Wang et al. placed self-efficacy between social support and anxiety or depression in patients with prostate cancer, whereas our model placed negative emotion before self-efficacy (Wang et al., 2022). Other postoperative models have examined psychological resources in relation to quality of life, rather than self-care confidence (Chen et al., 2025). The contribution of the present study is therefore to examine relational and emotional associations together in an early VATS recovery context, not to establish a definitive causal sequence. Repeated assessments are needed to distinguish the proposed ordering from alternative or reciprocal relationships.

Social support’s moderating role addresses a further question: whether the association between personal adaptive capacity and self-care confidence varies according to the available interpersonal resources. The resilience–self-efficacy association was positive at all three support levels, with slightly steeper slopes at higher support. Within COR theory, resources are embedded in social environments that can support or constrain their availability and use (Hobfoll et al., 2018; Hobfoll, 2001). A supportive environment therefore offers a plausible context for the observed interaction. This differs from the classical stress-buffering hypothesis, which concerns whether support weakens an adverse stress–well-being association (Cohen and Wills, 1985). Our interaction concerned resilience and support, not stress and support. Moreover, the small differences between the reported slopes warrant caution when judging clinical importance. The finding identifies a conditional association for further investigation; it does not demonstrate that increasing support would amplify resilience or initiate a resource-gain spiral.

For perioperative nursing, these findings suggest assessing self-care confidence together with patients’ adaptive capacity, emotional difficulties and available support. This psychosocial focus could complement ERAS research centred on physical recovery, such as Yang and Zheng’s chest-tube management study (Yang and Zheng, 2024). Assessment should remain individualised: the unadjusted urban–rural difference in self-efficacy was attenuated after adjustment and did not reach statistical significance, and residence alone cannot establish patients’ support needs. Existing intervention studies provide practical directions for evaluation. Gu et al.’s educational intervention improved pulmonary exercise compliance and illness perception, but not between-group frailty scores (Gu et al., 2025). Zhu et al.’s randomised trial found improvements in resilience, self-efficacy and quality of life following digital storytelling that combined psychological, social-support and health-behaviour content (Zhu et al., 2024). These findings make combined educational and psychosocial approaches reasonable candidates for further study, without establishing the indirect pathways or interaction observed here. Future evaluations should distinguish improvements in self-care confidence from changes in actual self-care behaviour and clinical recovery.

### 4.1 Limitations

Several limitations warrant consideration. The cross-sectional design means that the reported direct, indirect, and moderation effects should be interpreted as statistical associations rather than evidence of causal effects. In particular, the observed association between social support and the resilience–self-efficacy relationship cannot establish that enhancing social support would necessarily strengthen the role of resilience in self-efficacy. Longitudinal studies with repeated assessments and well-designed intervention studies are needed to establish temporal ordering and test this proposed causal chain. Recruitment from one tertiary hospital may limit generalizability, and the use of self-reported measures may have introduced recall or social-desirability bias.

## 5 Conclusions

In this study, intrinsic resilience was established as a dominant predictor of self-efficacy among lung cancer patients following VATS. We elucidated the complex psychosocial mechanisms underlying this relationship, demonstrating that it is partially and serially mediated by the sequential mobilization of social support and the subsequent reduction in negative emotions. Crucially, our findings reveal a profound synergistic moderating effect: abundant external social support significantly amplifies the protective effect of internal resilience on self-efficacy. Consequently, perioperative nursing care must evolve from isolated psychological counseling to comprehensive, systemic interventions. By simultaneously fostering resilience, mobilizing family and social networks, and mitigating negative emotions, nurses can synergistically empower post-VATS patients to achieve optimal self-management and recovery. Future longitudinal research is warranted to validate the causal pathways of these targeted, pathway-informed interventions.

## Ethics approval and consent to participate

Ethical approval for the study was obtained from the Ethics Committee at Sun Yat-sen University Cancer Center (Approval No.: SL-B2025–116–01), and all patients enrolled in the study were volunteered to sign the consent in person.

## Funding

None.

## CRediT authorship contribution statement

**Xinru Chen:** Writing – original draft, Formal analysis, Data curation, Conceptualization. **Gaoyang Duan:** Writing – original draft, Formal analysis, Data curation, Conceptualization. **Jiudi Zhong:** Supervision, Resources, Project administration, Data curation. **Heping Wu:** Supervision, Resources, Project administration, Data curation. **Yang Dong:** Writing – review & editing, Supervision, Formal analysis, Conceptualization. **Dandan Liu:** Writing – review & editing, Supervision, Formal analysis, Conceptualization.

## Declaration of competing interest

The authors declared no potential conflicts of interest with respect to the research, authorship, and/or publication of this article.

## Data Availability

Data and materials sharing are available from the corresponding authors on the condition of the reasonable reason.
