# Supplementary material for: Synergistic psychosocial pathways linking resilience to self-efficacy in lung cancer following video-assisted thoracic surgery: a cross-sectional study
Source: Int J Nurs Stud Adv. 2026 Oct 11;11:100705. doi: 10.1016/j.ijnsa.2026.100705 (PMC13694746; doi:10.1016/j.ijnsa.2026.100705)
Supplement: Supplementary file 1 [file mmc1.docx]

| **When submitting your paper, you must** submit a completed version of this checklist as your cover letter. You should also upload the checklist from any relevant reporting guideline (as supplemental material).  This checklist is intended to ensure that your manuscript meets some basic requirements for the journal. It should be read in conjunction with the guide for authors. Additionally, to help ensure your manuscript is compliant, a [manuscript template](https://legacyfileshare.elsevier.com/promis_misc/IJNS%20template.docx) is available. You should refer to it closely and we encourage you to use it to prepare your article. | | Insert a tick, page number(s) or give detail, where relevant |
| --- | --- | --- |
| **Template** | **I have used the manuscript template or checked that my manuscript is consistent with it** | √ |
| **Page numbers** | **The pages on the manuscript file and supplementary material are numbered** | √ |
| **Word count** | **The paper is 7000 words or fewer** | √ |
| **Tables and figures** | **I have included no more than five tables and figures in the main manuscript, and none exceed 1 A4 page in size / length** | √ |
|  | **Other tables and figures included in supplementary material are cited in the text** | √ |
| **Abbreviations** | **The body of my paper does not contain abbreviations** (other than SI units, names of measures or common statistical terms or exceptions in **universal** use, as detailed in the manuscript template).   - **No** abbreviations in title or abstract - Abbreviations in tables and figures are defined in a footnote to the table / figure | √ |
| **Reporting guideline** | **The paper has been prepared using a recognized reporting guideline appropriate to the method / type of paper and a copy of the associated checklist is included as supplemental material. Please identify the reporting guideline used in the box to the right.**   - Please consult <https://www.equator-network.org/> to help select an appropriate guide [research and reviews only]. | √ |
| **Title** | **The title is in the format ‘Topic or question: design or type of paper’** [not applicable to discussion papers or editorials] | √ |
| **Abstract** | **The paper includes a structured abstract of no more than 400 words**  Use headings appropriate to the design of the study. [Not applicable to editorials. Discussion paper abstracts can be unstructured] | √ |
| **Study registration** | **The abstract includes the study registration including number, the public registry**  **(e.g. ISRCTN), the registration date and date the first participant was recruited.** ^[1]^   - If not registered, state ‘not registered’^*^ | √ |
|  | **For clinical trials (as [defined by the ICMJE](https://www.icmje.org/about-icmje/faqs/clinical-trials-registration/)), registration occurred before the first participant was recruited.**   - We are unable to consider clinical trials that were registered retrospectively. | NA |
| **Key words** | **I have included keywords (maximum 7)**  Use the Medical Subject Headings or Cumulative Index to Nursing and Allied Health terms (see [http://www.nlm.nih.gov/mesh/meshhome.html](http://www.nlm.nih.gov/mesh/meshhome.html)). | √ |
| **Contribution of the Paper statements** | **I have included up to 6 article “highlights” formatted as short single sentence bullet points under the headings** “What is already known” and “What this paper adds” | √ |
| **Multiple publications** | **I have explicitly mentioned other publications arising from the same study / dataset as that used in my paper (see below)** | NA |
| **Ethical approval and informed consent** | **I have given details of the ethical approval, including the body that granted it and reference number at the end of my methods section.** | √ |
| **Statistical reporting** | **I have followed journal guidance on reporting statistics, confidence intervals and statistical significance (summarized below)** | √ |
|  | - Exact p-values are stated to an appropriate degree of precision (typically no more than 3 decimal points). - Where p values are reported, measures of effect or association and confidence interval are reported alongside all significance tests / p values (including in the abstract). - The term ‘statistically significant’ (not just ‘significant’) is used to refer to the result of tests. - p-values>0.05 (not statistically significant) are not interpreted as demonstrating no effect/association. - Where possible, we encourage you to use confidence intervals as the basis for inference without reference to statistical significance & p-values |  |
| **Qualitative findings** | **Key quotations to support inferences are given with give meaningful (anonymous) individual subject identifiers for each quotation used.** | √ |
| **Use of generative AI** | **I have reviewed the Elsevier [Generative AI policy](https://www.elsevier.com/about/policies-and-standards/generative-ai-policies-for-journals) and, if relevant, I have included a “Declaration of generative AI and AI-assisted technologies in the manuscript preparation process” before the reference list.** | NA |
| **Please provide below references for any other publications based on data from the same study that you report here** To assist editors, upload copies of papers where the abstract / full text is not readily available (including those under review elsewhere, which will be treated in strict confidence).  Other published and in press accounts of the study from which data in this paper originate must be referred to in the paper (author details can be redacted for review if desired) and the relationship between this and other publications from the same study must be made clear in the paper.  e.g. “A previous publication from this study showed that carrots were effective in preventing blindness. This study reports on a range of other health benefits, which were not reported in our earlier paper [give full reference / doi link].”  Where your paper is based on analysis of a publically available data set or is part of a series of publications from a large cohort study (or similar) you can be selective in the references you provide and give a more general account of how this paper relates to others but it is essential that editors are able to verify the unique contribution of the paper you are submitting.  If unsure about declarations we encourage you to err on the side of openness and suggest you consult Norman, I., Griffiths, P., 2008. Duplicate publication and 'salami slicing': Ethical issues and practical solutions. International Journal of Nursing Studies 45 (9), 1257-1260 [doi 10.1016/j.ijnurstu.2008.07.003](https://doi.org/10.1016/j.ijnurstu.2008.07.003). | | |
| Insert details of previous publications *from this study* below. **Describe the relationship to the current study**  NA | | |

*© Peter Griffiths, Ian Norman – 2026 (April)*

*This work is licensed under a* [Creative Commons Attribution 4.0 International License.](http://creativecommons.org/licenses/by/4.0/)

1. While the journal endeavours to maintain a double blind-review process as far as possible, we give priority to transparent reporting and prospective registration. As it is important that reviewers can verify that reporting is complete and consistent with protocols to avoid (for example) selective outcome reporting or undocumented protocol changes, authors are not permitted to redact registration numbers for review. By choosing to submit you acknowledge this exception to anonymity in the review process.
